# Enhanced isolation of SARS-CoV-2 by TMPRSS2-expressing cells

**DOI:** 10.1073/pnas.2002589117

**Published:** 2020-03-12

**Authors:** Shutoku Matsuyama, Naganori Nao, Kazuya Shirato, Miyuki Kawase, Shinji Saito, Ikuyo Takayama, Noriyo Nagata, Tsuyoshi Sekizuka, Hiroshi Katoh, Fumihiro Kato, Masafumi Sakata, Maino Tahara, Satoshi Kutsuna, Norio Ohmagari, Makoto Kuroda, Tadaki Suzuki, Tsutomu Kageyama, Makoto Takeda

**Affiliations:** ^a^Department of Virology 3, National Institute of Infectious Diseases, Tokyo 162-8640, Japan;; ^b^Influenza Virus Research Center, National Institute of Infectious Diseases, Tokyo 162-8640, Japan;; ^c^Department of Pathology, National Institute of Infectious Diseases, Tokyo 162-8640, Japan;; ^d^Pathogen Genomics Center, National Institute of Infectious Diseases, Tokyo 162-8640, Japan;; ^e^Disease Control and Prevention Center, National Center for Global Health and Medicine, Tokyo 162-8655, Japan

**Keywords:** coronavirus, SARS-CoV-2, VeroE6, outbreak, TMPRSS2

## Abstract

A novel betacoronavirus, severe acute respiratory syndrome coronavirus 2 (SARS-CoV-2), which caused a large respiratory outbreak in Wuhan, China in December 2019, is currently spreading across many countries globally. Here, we show that a TMPRSS2-expressing VeroE6 cell line is highly susceptible to SARS-CoV-2 infection, making it useful for isolating and propagating SARS-CoV-2. Our results reveal that, in common with SARS- and Middle East respiratory syndrome-CoV, SARS-CoV-2 infection is enhanced by TMPRSS2.

In December 2019 a respiratory outbreak from a novel betacoronavirus, severe acute respiratory syndrome coronavirus 2 (SARS-CoV-2), occurred in Wuhan City, China (1, 2). As of 9 February 2020, 37,558 confirmed cases and 813 deaths had been recorded. Although the great majority of cases were reported in China, 24 countries had already been affected with 307 confirmed cases. On 15 January 2020, the first case was detected in Japan. As of 10 February 2020, Japan had 16 domestically confirmed cases and 9 returnees from Wuhan using government-chartered flights. In addition, 70 cases were confirmed on a SARS-CoV-2–quarantined cruise ship.

SARS-CoV-2 is isolatable using VeroE6, Huh7, or human airway epithelial cells (2–4), but here we show that an engineered cell line, VeroE6/TMPRSS2, is highly susceptible to SARS-CoV-2 infection, suggesting the important role for TMPRSS2 in SARS-CoV-2 infection and indicating its potential utility for isolating and propagating this virus.

Previous studies (5, 6) have shown that the phylogenetically related SARS-CoV is proteolytically activated by TMPRSS2 in vitro and in vivo. Therefore, we attempted to isolate SARS-CoV-2 using VeroE6/TMPRSS2 cells, which express TMPRSS2 constitutively. The messenger RNA expression level of TMPRSS2 in VeroE6/TMPRSS2 cells is ∼10-fold higher than in normal human lung tissue and other human cell lines (Fig. 1*A*). SARS-CoV-2 uses the same receptor, ACE2, as SARS-CoV (2), and ACE2 expression is very high in VeroE6 cells (7). Seven clinical specimens (throat swabs or sputum) obtained from seven SARS-CoV-2 infection cases were inoculated into VeroE6/TMPRSS2 cells, which were monitored daily for cytopathic effect (CPE). These clinical specimens were deidentified prior to use, and this study was approved by the ethics committee of the National Institute of Infectious Diseases, Japan (approval no. 1091). Informed consent was obtained from all participants, from which the subjects were obtained, or their legally acceptable representatives for sample donation. In five cases among the seven, clear CPE with detachment/floating (black arrows, Fig. 1*B*) and syncytium formation (white arrows, Fig. 1*B*) developed at 2 or 3 d postinfection (p.i.) (Table 1). The virus titers in culture supernatants of the five cases at 3 d p.i. were 4.6 × 10^6^ to 6.8 × 10^7^ median tissue culture infectious dose (TCID_50_) per mL (Table 1). Typical coronavirus particles were detected by electron microscopy (Fig. 1*C*). Next-generation sequencing (NGS) of case Wk-521 detected the nearly full-length genome sequence from SARS-CoV-2 with >99.9% homology (1, 2) (GISAID database ID EPI_ISL_408667). Unexpectedly, the NGS data showed contaminated mycoplasma sequences (*Mycoplasma hyorhinis* and *Mycoplasma arginini*) from VeroE6/TMPRSS2 cells. CPE in VeroE6 cells persistently infected with SARS-CoV was enhanced by infection with *Mycoplasma fermentans* (8), but whether a similar situation exists for SARS-CoV-2–related CPE in this cell line is unclear.

**Fig. 1.**
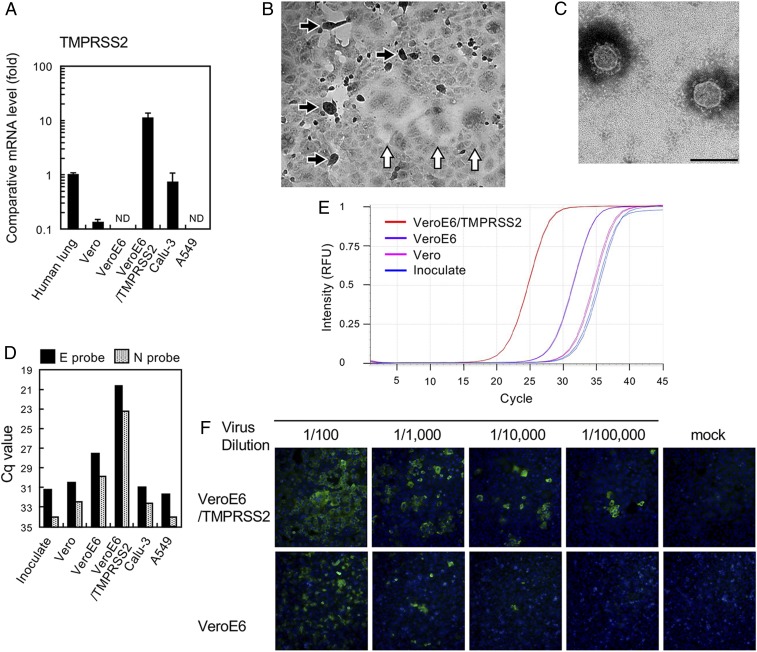
(*A*) Expression of TMPRSS2 in total cellular RNA (0.2 µg) of indicated cells was compared with that in human lung RNA (catalog no. 636524; Clontech) by quantitative real-time PCR. ND, not detectable. (*B*) SARS-CoV-2–infected VeroE6/TMPRSS2 cells. Cell rounding (black arrows) and syncytium formation (white arrows) (*C*). Electron micrograph showing isolated virus particles with negative staining. (Scale bar, 200 nm.) (*D*) Viral RNA multiplication in various cells at 48 h postinoculation with the viral specimen, as determined by real-time RT-PCR using E and N primer/probe sets (9). Cq, quantitation cycle. (*E*) Real-time RT-PCR amplification plot using the E primer/probe set, corresponding to the data in *C*. RFU, relative fluorescence units. (*F*) Comparison of cell susceptibility to the isolated virus, detected with a patient’s serum and Alexa 488-conjugated goat anti-human IgG. Nuclei were stained with DAPI.

**Table 1.** SARS-CoV-2 cases and virus isolation

	Cq value of specimens				
Case	N set*	N2 set*	Virus isolation	Days (CPE appearance)	Virus titer at 3 d p.i., TCID_50_/mL
V-009	33.87	30.87	No	>6 d	u.d.
Wk-177	u.d.	35.08	No	>6 d	u.d.
I-004	34.52	31.66	Yes	3 d	2.2 × 10^7^
V-029	32.85	28.80	Yes	3 d	4.6 × 10^6^
Wk-012	33.53	29.60	Yes	3 d	2.2 × 10^7^
Wk-501	27.35	21.76	Yes	2 d	6.8 × 10^6^
Wk-521	29.68	24.41	Yes	2 d	6.8 × 10^7^

u.d.: undetermined.

* The real-time RT-PCR primers and probe sets (N and N2 sets) are described in Shirato et al. (10).

The viral RNA copies in the clinical specimens used for virus isolation were estimated by real-time RT-PCR (9, 10). As expected, viral RNA copies in the clinical specimens in which CPE developed within 2 d p.i. were greater than those in the other specimens (Table 1).

VeroE6/TMPRSS2 cells are superior to other cell lines tested in this study for SARS-CoV-2 isolation. Consistent with previous reports (2, 4), the amount of SARS-CoV-2 RNAs in the culture supernatants of Vero, Calu-3, and A549 cells 48 h p.i. was low and was measurably higher when VeroE6 cells were used. However, the viral RNA copies in the VeroE6/TMPRSS2 cell culture supernatants were >100 times greater than those from VeroE6 cells (Fig. 1 *D* and *E*). Data for SARS-CoV show that TMPRSS2 enhances its entry efficiency (5, 11). VeroE6 and VeroE6/TMPRSS2 cells were infected with 10-fold serially diluted SARS-CoV-2 samples, and the infected cells were visualized by indirect immunofluorescent assays (Fig. 1*E*). The results showed that VeroE6/TMPRSS2 displayed ∼10-fold greater number of SARS-CoV-2–infected cells than the parental VeroE6 cells. These data suggest that, in common with SARS-CoV, TMPRSS2 may also play an important role in SARS-CoV-2 cell entry.

SARS-CoV and Middle East respiratory syndrome (MERS)-CoV can enter cells via endocytosis and use cathepsin in endosomes for activation (12–14). However, TMPRSS2 expression greatly promotes replication and syncytium formation in these viruses in vitro and in vivo (5, 6, 11, 12, 15). Our findings suggest that TMPRSS2 is also likely to be a key protease for SARS-CoV-2 replication. Thus, developing TMPRSS2-related therapeutic agents may be a promising countermeasure against the current and new outbreaks of CoVs.

TMPRSS2-expressing cell lines are highly susceptible to SARS-CoV, MERS-CoV, and SARS-CoV-2, making the VeroE6/TMPRSS2 cell line a suitable contributor to the global surveillance of high-risk CoVs. VeroE6/TMPRSS2 cells are easily maintained, suitable for large-scale propagation, and now available from Japanese Collection of Research Bioresources (JCRB) Cell Bank in Japan (https://cellbank.nibiohn.go.jp/english/) (JCRB no. JCRB1819). Treatment for mycoplasma is now ongoing in the JCRB Cell Bank.

## Data Availability

Data have been deposited in the Global Initiative on Sharing All Influenza Data (GISAID) database with accession ID EPI_ISL_408667. The cell line information is available from JCRB Cell Bank in Japan (https://cellbank.nibiohn.go.jp/english/) (JCRB no. JCRB1819).
